# Metabarcoding Reveals Cover Crop Functional Type as Primary Driver of Soil Nematode Community Composition in a Carrot Production System

**DOI:** 10.3390/biology15151226

**Published:** 2026-07-23

**Authors:** Syed Atif Hasan Naqvi, Jerry Akanwari, Tahera Sultana

**Affiliations:** 1 Department of Biological Sciences, Brock University, St. Catharines, ON L2S 3A1, Canada; na24jz@brocku.ca (S.A.H.N.); ja20yb@brocku.ca (J.A.); 2 London Research and Development Center, Agriculture and Agri-Food Canada, Vineland Station, Lincoln, ON L0R 2E0, Canada

**Keywords:** maturity index, channel index, soil food web, legume–non-legume covers mixture, ecological indices, nematode feeding groups

## Abstract

The influence of cover crop functional type on soil nematode feeding group structure, food web condition, and crop productivity in horticultural systems remains poorly understood. Using DNA metabarcoding targeting the V6–V8 region of the 18S rRNA gene, we characterized nematode community responses in soil samples collected from a carrot (*Daucus carota* L.) production system in Ontario, Canada. We evaluated the effects of alfalfa (*Medicago sativa* L.), sorghum (*Sorghum bicolor*), and their mixture under two nitrogen regimes. Bacterivores dominated all treatments (60.8%), whereby sampling time and cover crop identity jointly explained 33.5% of community variance. Nematode community ecological indices revealed highly significant temporal shifts in food web condition, including seasonal decline in maturity and structure indices and a rise in the channel index, indicating food web restructuring regardless of cover crop type. Individual cover-crop exhibited distinct trade-offs between nematode suppression and crop productivity, while sorghum drove a pronounced bacterivore-to-fungivore transition. The alfalfa–sorghum (legume–non-legume) mixture emerged as the most agronomically balanced treatment, uniquely combining plant-parasitic nematode suppression with the highest individual carrot weight and the most balanced soil food web structure. This study provides important insights into how cover crop functional type influences soil nematode communities and crop performance in carrot production systems. Future studies repeating this experiment across multiple locations and growing seasons would help determine the consistency of these responses under a broader range of environmental conditions.

## Simple Summary

Soil health is the foundation of productive farming; however, its association with soil microbes is not fully understood. Cover crops are plants grown between cash crop seasons to protect and improve soil health. However, their effects on soil food webs remain poorly understood. The study evaluated legume (alfalfa) and non-legume (sorghum) cover crops, as well as their mixture, in a carrot production system at the Jordan Research farm, Ontario, Canada. This research used DNA-based methods to study nematode communities that changed across the growing season. After the cover crops were incorporated into the soil, half of the plots received nitrogen fertilizer, while the remaining plots did not. This allowed us to assess how nitrogen influenced nematode communities and carrot performance. The cover crop mixture provided the most balanced agronomic outcome, combining moderate nematode suppression with the highest individual carrot weight and the most balanced soil food web structure. Ecological indices revealed a significant seasonal restructuring of soil food webs across all treatments. These findings demonstrate that while individual cover crops present trade-offs between pest suppression and yield, functionally diverse cover crop mixture offers the most practical strategy for farmers aiming to simultaneously enhance soil health, suppress plant-parasitic nematodes, and sustain carrot yield and quality.

## 1. Introduction

Soil nematodes are among the most abundant and diverse metazoans on Earth, playing fundamental roles in soil food webs and ecosystem functioning [1]. While most soil nematodes are beneficial free-living organisms that contribute to nutrient cycling and decomposition processes, plant-parasitic nematodes (PPNs) represent a significant threat to global food security, causing estimated annual crop losses of USD 80–173 billion worldwide [2]. In tropical and subtropical regions, crop production losses due to nematode infestation average 14.6%, compared to 8.8% in temperate climates [3]. Because nematode damage often occurs belowground, and its symptoms often resemble nutrient deficiencies or water stress, its economic impact is frequently underestimated [4]. With increasing restrictions on chemical nematicides due to environmental and human health concerns, there is an urgent need for sustainable, ecologically based management strategies that can simultaneously suppress plant-parasitic nematodes while promoting beneficial soil organisms [5].

Nematode communities have emerged as powerful bioindicators of soil health and ecosystem functioning due to their well-characterized taxonomy, diverse feeding habits, and differential sensitivity to environmental disturbance [6]. Free-living nematodes occupy multiple trophic levels within soil food webs, including bacterivores, fungivores, omnivores, and predators, each responding differently to changes in soil conditions and management practices [7]. Omnivorous and predatory nematodes, though typically comprising a smaller proportion of total nematode communities (5–15% and 1–5%, respectively), play disproportionately important roles in regulating food web stability by controlling lower trophic-level populations and redistributing nutrients across trophic levels, thereby enhancing overall soil food web resilience [6,7]. The ratio of bacterial-feeding to fungal-feeding nematodes reflects the dominant decomposition pathway: bacterial dominance indicates rapid nutrient cycling through the bacterial energy channel, while fungal dominance suggests slower, more conservative nutrient turnover through the fungal channel [8]. The development of ecological indices based on nematode community structure, including the maturity index (MI), enrichment index (EI), structure index (SI), channel index (CI), and basal index (BI), has provided standardized tools for assessing soil food web condition across diverse agroecosystems [9]. These indices integrate information about nematode life-history strategies, trophic interactions, and food web complexity into quantitative measures of ecosystem condition that are stable across environments and more ecologically informative than simple diversity metrics such as the Shannon index [10,11].

Cover cropping has gained increasing attention as a sustainable agricultural practice that can simultaneously improve soil health, suppress pests, and enhance ecosystem services [12,13,14]. Cover crops influence soil nematode communities through multiple mechanisms, including the provision of organic matter inputs, modification of soil physical and chemical properties, and direct effects on nematode populations through host–parasite interactions or allelopathic compounds [15,16]. Recent studies have demonstrated that cover cropping generally promotes free-living nematode populations while reducing plant-parasitic nematodes, with effects varying substantially by cover crop functional type and residue quality; for instance, studies in oak tree systems [17] and brassica and rye crop systems [18] have shown considerable variation in nematode community responses depending on the functional type of cover crop employed. Cover crop residues play a critical role in determining decomposition pathways and subsequent effects on soil biota [19]. Residues from legumes decompose rapidly through bacterial pathways, stimulating bacterivore nematodes, while residues from non-legume covers favor fungal decomposition and associated fungivore nematodes, collectively shaping the structure of soil nematode communities [20,21,22,23,24,25].

Nitrogen availability represents another critical factor modulating cover crop effects on soil health. Nitrogen fertilization can alter the abundance and composition of microbial food resources and plant root quality [26]. High nitrogen inputs generally favor bacterial-feeding nematodes and may increase plant-parasitic nematode populations due to enhanced root growth and quality [27]. Conversely, nitrogen limitation can shift decomposition toward fungal-dominated pathways and potentially reduce plant-parasitic nematodes pressure [20]. The interaction between cover crop type and nitrogen management on nematode communities, however, remains poorly understood despite its potential importance for developing integrated soil health management strategies. Most previous studies in this domain have focused on large-scale field crops such as corn and soybean [17,18], with comparatively limited investigation of cover crop–nematode interactions in horticultural vegetable production systems such as carrot, spinach, or tomato, where economic thresholds for nematode damage are generally lower and pest management decisions are more sensitive to biological control efficacy. Furthermore, most previous studies have relied on single time-point assessments, limiting the understanding of how nematode communities respond temporally across the cover crop-incorporation and cash crop-growing period [28].

Carrot (*Daucus carota* L.) production is particularly vulnerable to plant-parasitic nematode damage, as root-feeding nematodes directly compromise the marketable production. While the soil health benefits of cover crops are increasingly well-documented, their agronomic trade-offs, particularly their effects on subsequent crop yield, remain less consistently characterized [5]. Cover crops influence carrot productivity through multiple pathways including nitrogen cycling, soil structure modification, allelopathic residue effects, and the modification of soil biotic communities including nematode populations [6]. Understanding yield trade-offs associated with nematode-suppressive cover crops is therefore essential for evaluating their practical value in commercial carrot production systems. Alfalfa was selected as the legume cover crop due to its well-documented capacity as a non-host or poor host for economically important plant-parasitic nematodes, its ability to promote beneficial soil microbiomes associated with nematode suppression, and its widespread adoption in Ontario horticultural systems [29]. Sorghum was selected as the non-legume component due to its high biomass production, characteristically high C:N ratio residues that promote fungal decomposition pathways, and documented allelopathic potential against plant-parasitic nematodes through dhurrin-derived compounds [29]. This study evaluated the effects of these contrasting cover crop functional types, individually and in mixture, on soil nematode communities and assesses their interactive effects with nitrogen fertilization in carrot production. Understanding these dynamics is essential for developing cover crop-based management strategies that simultaneously enhance soil health indicators and suppress plant-parasitic nematode populations in horticultural production systems.

## 2. Materials and Methods

### 2.1. Site Management and Soil Sampling

The research was conducted at Jordan Research Station (43.1615° N, 79.3676° W), Agriculture and Agri-Food Canada, ON, Canada. The experiment was arranged in a randomized complete block design (RCBD) with a split-plot arrangement. The total experimental field measured approximately 20 m × 25 m, consisting of three cover crop treatments and one control site with four replications. Each plot measured 1.5 m × 8.5 m with a 3 m buffer zone between plots. The three cover crop treatments were as follows: alfalfa (*Medicago sativa* L.), sorghum (*Sorghum bicolor*), a 50:50 mixture of alfalfa and sorghum; and a control (no cover crop). Plots were subdivided into two subplots (1.5 m × 4 m each) with a 0.5 m buffer between subplots to minimize treatment interference while nitrogen was implemented. Each subplot received one of two nitrogen treatments: synthetic nitrogen fertilization (120 kg N ha^−1^, applied at carrot seeding as per growers’ recommendation) or no nitrogen application (control). A schematic representation of the experimental plot layout is provided in Supplementary Figure S1.

Cover crops were established following regional seeding rate recommendations optimized for organic soil conditions [29]: alfalfa at 15 kg ha^−1^ and sorghum at 45 kg ha^−1^ as monocultures, and the mixture treatment at 7.5 kg ha^−1^ alfalfa and 22.5 kg ha^−1^ sorghum. Cover crops were grown until maturity and incorporated into the soil in late June using standard seedbed preparation consisting of moldboard plowing and secondary tillage with a field cultivator, allowing initial biomass decomposition prior to carrot planting. Carrot (*Daucus carota* L., cultivar ‘Gold Finger’) seeds were planted 14 days after cover crop incorporation allowing sufficient time for biomass incorporation. Seeds were sown at 2 cm spacing within rows. Carrots were grown for approximately 49 days, corresponding to the typical maturity period for the cultivar. Weed control and other crop management practices were performed according to standard farm management procedures.

Soil samples were collected at two time points to capture temporal dynamics in nematode communities: (i) before cover crop incorporation (late June; hereafter cover crop stage) and (ii) at carrot harvest (early September; hereafter harvest). At each sampling time, ten soil cores were collected per plot using a 2.5 cm diameter soil probe to a depth of 20 cm, following a systematic W-pattern across the plot to ensure representative spatial coverage. The 10 cores from each plot were combined to form a single composite sample. A total of 48 composite samples were collected from both sampling stages (16 at the cover crop stage and 32 at harvest). Soil samples were placed in sealed polyethylene bags, stored in coolers with ice packs (maintained at 4 °C), and transported to the laboratory immediately. Upon arrival, samples were gently homogenized, large debris (stones, roots > 5 mm diameter) were removed, and stored at 4 °C for further processing.

### 2.2. DNA Extraction, Sequencing and Bioinformatics Analysis

Nematodes were extracted from 100 g of each soil replicate using the centrifugation and sugar flotation [30] method. Prior to DNA extraction, extracted nematodes were submerged in liquid nitrogen for five seconds and DNA was extracted using the DNeasy Blood & Tissue Kit (Cat: 69504, Qiagen Inc., Mississauga, ON, Canada) following the manufacturer’s instructions. Extracted DNA was quantified using a Nanodrop (NanoDrop 2000, Thermo Fisher Scientific, Waltham, MA, USA). Final DNA concentrations ranged from 15.3 to 89.7 ng µL^−1^ (mean: 42.8 ± 18.4 ng µL^−1^). An average of 20 ng µL^−1^ DNA per sample was submitted to Génome Québec (Montréal, QC, Canada) for PCR amplification, library preparation, and paired-end sequencing on an Illumina MiSeq platform (2 × 300 bp). Amplification targeted the V6–V8 region of the 18S rRNA gene using primers NF1 (5′-GGTGGTGCATGGCCGTTCTTAGTT-3′) and 18Sr2b (5′-TACAAAGGGCAGGGACGTAAT-3′), which represent the most widely used primer pair for nematode metabarcoding studies.

Raw sequence data were uploaded to the AAFC cluster of the Digital Research Alliance of Canada. The fastp plugin was used to remove primers and low-quality reads. Forward and reverse reads were trimmed to 280 bp and 270 bp, respectively, with a maximum *N* = 0 and maximum expected error (EE) = 2. The nf-core/ampliseq Nextflow pipeline (version 2.7.1) was used to process trimmed reads, applying a minimum 10 bp overlap for paired-end merging. Chimeric sequences were identified and removed [31]. Raw paired-end sequencing reads generated in this study have been deposited in the NCBI Sequence Read Archive (SRA) under BioProject accession number PRJNA1492785. OTUs with total reads representing <0.01% of the total dataset were filtered to remove potential sequencing artifacts and low-abundance contaminants. Taxonomy was assigned to OTUs using a Naive Bayes q2-feature-classifier trained on the NemaTaxa database [32] at a 99% species identity threshold, consistent with benchmarks recommended for nematode metabarcoding studies. Nematode OTUs were assigned to trophic feeding groups, namely bacterivores, fungivores, herbivores (plant-parasitic nematodes), omnivores, and predators following established classification frameworks [33]. The relative abundance of each feeding group was calculated as the proportion of total OTUs per sample assigned to that group, expressed as a percentage. Nematode community ecological indices including the Maturity Index (MI), Channel Index (CI), and Structure Index (SI), were calculated using established equations as described in Section 2.3, based on colonizer–persister (*c-p*) values assigned to free-living nematode genera [11,34]. 

### 2.3. Nematode Community Analysis

Nematode community ecological indices were calculated to characterize soil food web condition. The maturity index (MI) was computed as the weighted mean of colonizer–persister (*c-p*) values for all free-living nematode genera (excluding plant-parasitic nematodes), where c-p values range from one (extreme colonizers: short life cycle, high fecundity, disturbance-tolerant) to five (extreme persisters: long life cycle, low fecundity, disturbance-sensitive) [11,33,34], following Equation (1):

(1) $$MI=\sum_{i=1}^{n}v_{i}\cdot f_{i}$$

where *v_i_* is the *c-p* value of genus *i* and *f*_i_ is the relative frequency (proportional abundance) of genus *i* among free-living taxa.

The enrichment index (EI), structure index (SI), basal index (BI), and channel index (CI) were computed following the functional guild framework of [10], in which each nematode genus is classified by both feeding type and (*c-p*) value into functional guilds. Guild-specific indicator weights (k) were applied to nematode abundance (*n*) to calculate weighted enrichment (*e*), structure (*s*), and basal (*b*) components as defined in Equation (2):

(2) $$e=\sum k_{e}\cdot n_{e}\;\;s=\sum k_{s}\cdot n_{s}\;\;b=\sum k_{b}\cdot n_{b}$$

The four indices were then derived from these components as given in Equations (3)–(6):

(3) $$EI=100\times \frac{e}{e+b}$$

(4) $$SI=100\times \frac{s}{s+b}$$

(5) $$BI=100\times \frac{b}{e+s+b}$$

(6) $$CI=100\times \frac{{Fu}_{2}\times 0.8}{{Ba}_{1}\times 3.2+{Fu}_{2}\times 0.8}$$

where *Ba*_1_ and *Fu*_2_ represent the abundances of opportunistic bacterivores (*c-p*_1_) and opportunistic fungivores (*c-p*_2_), respectively, and 3.2 and 0.8 are their enrichment weights.

The EI reflects resource availability and nutrient enrichment; the SI reflects food web structural complexity and higher trophic connectivity; the BI represents the proportion of basal, stress-tolerant taxa; and the CI indicates the relative contribution of fungal versus bacterial decomposition pathways (CI > 50 indicates fungal-dominated decomposition). Food web condition was visualized by plotting treatment group means ± SE for EI and SI in the four-quadrant diagnostic framework, with quadrant boundaries at EI = 50 and SI = 50. Colonizer–persister values for each genus were assigned based on published classifications [35].

### 2.4. Statistical Analysis and Data Visualization

All statistical analyses and data visualizations were conducted in R v4.3.1 [36] using the vegan and ggplot2 [37] packages, with significance threshold set at α = 0.05. Two-way ANOVA was used to test treatment effects on feeding groups, ecological indices, and carrot yield, followed by Tukey’s HSD (*p* < 0.05); data were transformed (square-root or log, x + 1) where required to meet model assumptions. Nematode community alpha-diversity was calculated from rarefied OTU abundance data, and beta-diversity was assessed using Bray–Curtis dissimilarity indices. Non-metric multidimensional scaling (NMDS) was performed to visualize community structure patterns across treatments and sampling times, with ordination stress values <0.15. Redundancy Analysis (RDA) was used to quantify the variation in nematode community composition explained by cover crops and nitrogen treatments at harvest, with significance tested by permutation testing (9999 permutations).

## 3. Results

### 3.1. Nematode Feeding Group Composition

**Figure 1:**
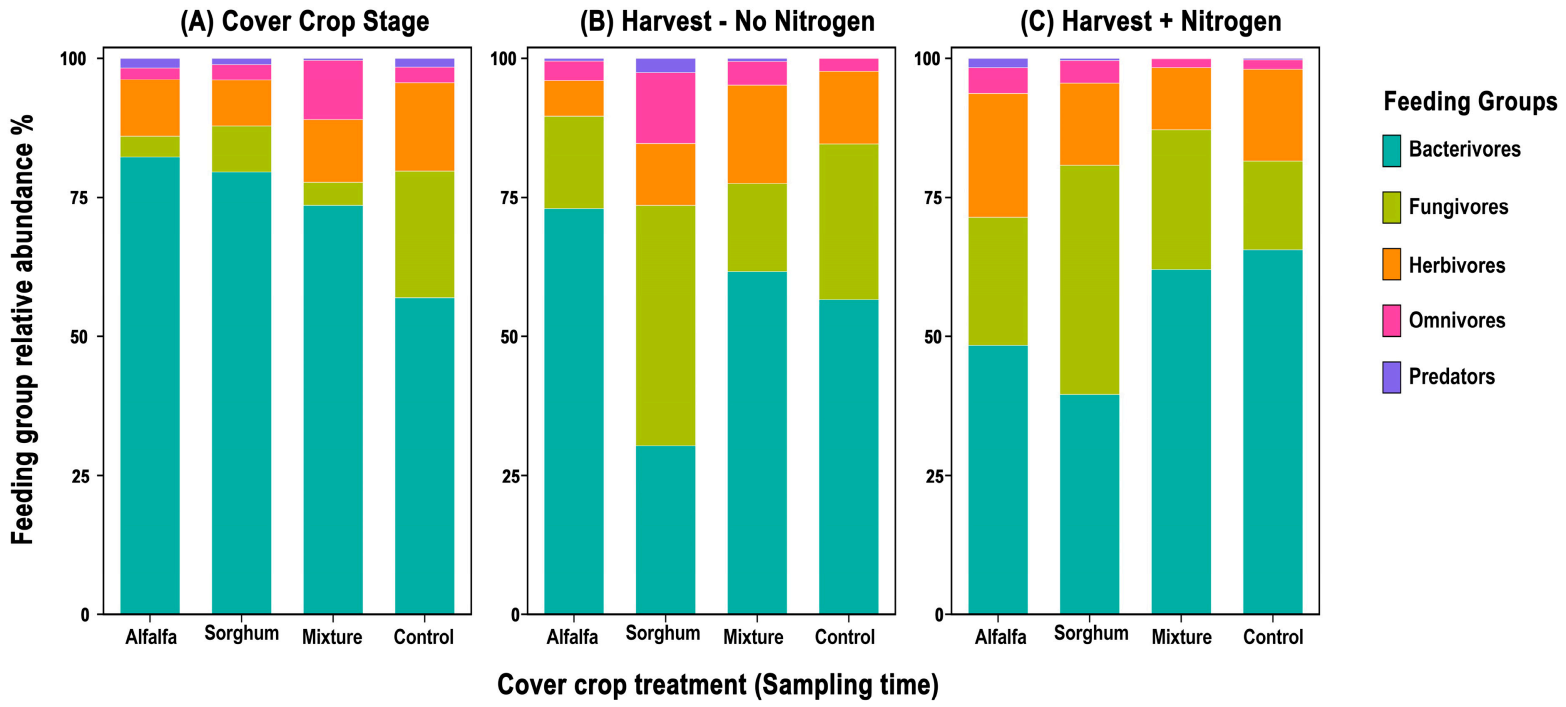
Relative abundance (%) of nematode feeding groups across cover crop treatments and sampling periods in carrot. (**A**) Cover crop stage (post-incorporation); (**B**) harvest without nitrogen fertilization; (**C**) harvest with nitrogen fertilization. Stacked bars represent the mean relative abundance of five feeding groups: bacterivores, fungivores, herbivores (plant-parasitic nematodes), omnivores, and predators. Cover crop treatments include alfalfa, sorghum, mixture (sorghum + alfalfa), and control (no cover-crop). This figure presents community composition descriptively; and ANOVA results for individual feeding group abundances across cover crop treatments, sampling time, nitrogen, and their interactions are reported in Table S1.

Nematode feeding group composition was determined by assigning OTUs to trophic groups. Across all 48 samples, the community was dominated by bacterivores (60.8%), followed by fungivores (20.4%), plant-parasitic nematodes (herbivores; 13.3%), omnivores (4.6%), and predators (0.9%). Feeding group composition varied across both sampling time and cover crop treatment. At the cover crop stage, bacterivore dominance was highest in alfalfa (82%) and sorghum (80%) plots, while control plots showed a reduced bacterivore proportion (57%) with a corresponding increase in fungivores and omnivores. At harvest without nitrogen, sorghum plots exhibited a marked decline in bacterivore abundance (30%) with a corresponding increase in fungivores (45%), indicating that sorghum residues promoted fungal over bacterial decomposition. Alfalfa plots maintained elevated bacterivore proportions under both nitrogen regimes at harvest (72% without nitrogen; 48% with nitrogen). Mixture plots exhibited intermediate community composition throughout the growing season, reflecting the combined influence of legume and non-legume residue inputs (Figure 1). Sampling time significantly affected bacterivore abundance (*p* = 0.003, partial η^2^ = 0.183), with communities shifting from bacterivore-dominated at the cover crop stage to more evenly distributed at harvest. Cover crop type significantly affected fungivore abundance (*p* = 0.045, partial η^2^ = 0.172), driven primarily by sorghum plots. Nitrogen fertilization did not independently affect any feeding group (*p* > 0.05); however, a significant alfalfa × nitrogen interaction was detected for plant-parasitic nematodes (*p* = 0.027), confirming that nitrogen fertilization differentially modified the herbivore-suppressive effect of alfalfa residues (Table S1A).

### 3.2. Genus-Level Community Composition

**Figure 2:**
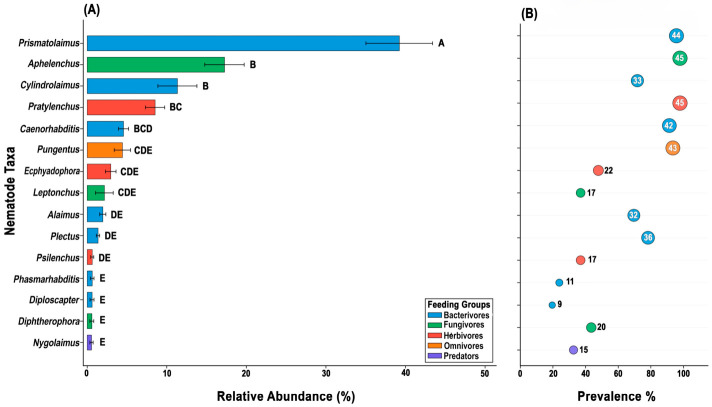
Mean relative abundance (±SE) and the prevalence of the 15 most abundant nematode genera identified across all samples. (**A**) Horizontal bar chart showing mean relative abundance (%) with standard error bars; genera are ranked from highest to lowest abundance and color-coded by five feeding types. Letters indicate significant differences in mean relative abundance among the 15 genera, compared against each other using a Kruskal–Wallis test followed by Dunn’s post hoc pairwise comparison with Holm correction (α = 0.05); genera sharing the same letter are not significantly different. (**B**) Prevalence expressed as the number of samples in which each genus was detected; bubble size is proportional to detection frequency.

The nematode community was dominated by 15 genera accounting for the majority of total relative abundance. *Prismatolaimus* (bacterivore) was the most abundant genus, representing a mean relative abundance of 39.2% across samples (detected in 95.7% of samples). *Aphelenchus* (fungivore) and *Pratylenchus* (plant-parasitic nematode) were the most consistently detected genera, present in 97.8% samples, with mean relative abundances of 16.7% and 8.9%, respectively. Bacterivorous genera collectively dominated the top 15, with seven genera (*Prismatolaimus*, *Cylindrolaimus*, *Caenorhabditis*, *Alaimus*, *Plectus*, *Phasmarhabditis*, *Diploscapter*) representing this feeding group (Figure 2).

**Figure 3:**
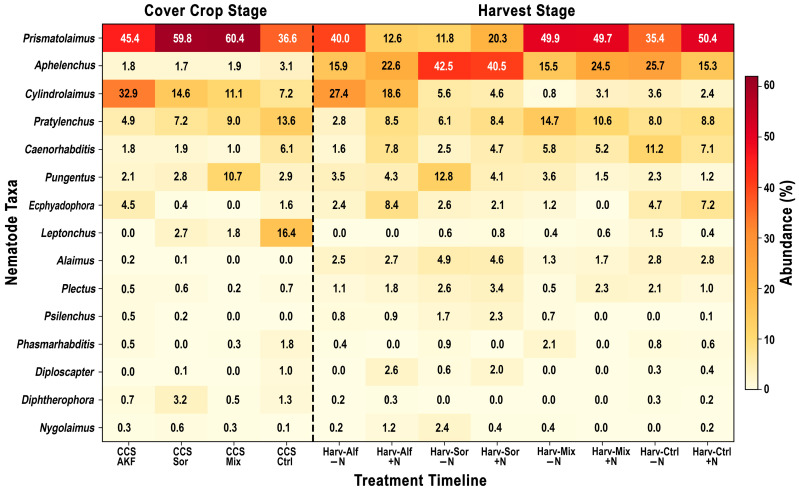
Heatmap of mean relative abundance (%) of the 15 most abundant nematode genera across cover crop treatments and sampling periods. Columns represent treatment combinations at two sampling times: cover crop stage (CCS; alfalfa, sorghum, mixture, control) and harvest (with and without nitrogen fertilization for each cover crop). Rows are ordered by overall mean abundance. Color intensity reflects abundance from low (pale yellow) to high (dark red). The dashed vertical line separates cover crop stage from harvest. Values within cells represent treatment-level means. No pairwise statistical comparisons are displayed; this figure presents genus-level compositional patterns descriptively across all treatment–time combinations.

Heatmap analysis of the 15 most abundant genera revealed pronounced genus-level shifts in community composition across treatment and time. *Prismatolaimus* reached its highest relative abundance in cover crop stage sorghum (59.8%) and mixture (60.4%) plots but showed variable responses across harvest treatments. *Aphelenchus* (fungivore) showed the most dramatic temporal shift, increasing from low levels at the cover crop stage (1.7–3.1%) to 42.5% in harvest sorghum plots without nitrogen, reflecting the progressive development of fungal decomposition pathways. *Cylindrolaimus* (bacterivore) was notably abundant at the cover crop stage under alfalfa (32.9%) but declined across all treatments by harvest. *Pratylenchus* (plant-parasitic nematodes) maintained relatively stable abundance across most treatment–time combinations (2.8–14.7%), with the lowest values in alfalfa harvest plots. Together, these genus-level patterns reveal that the broad feeding group shifts were driven by differential temporal responses of specific genera rather than uniform community-wide reorganization (Figure 3).

### 3.3. Nematode Community Ecological Indices

**Figure 4:**
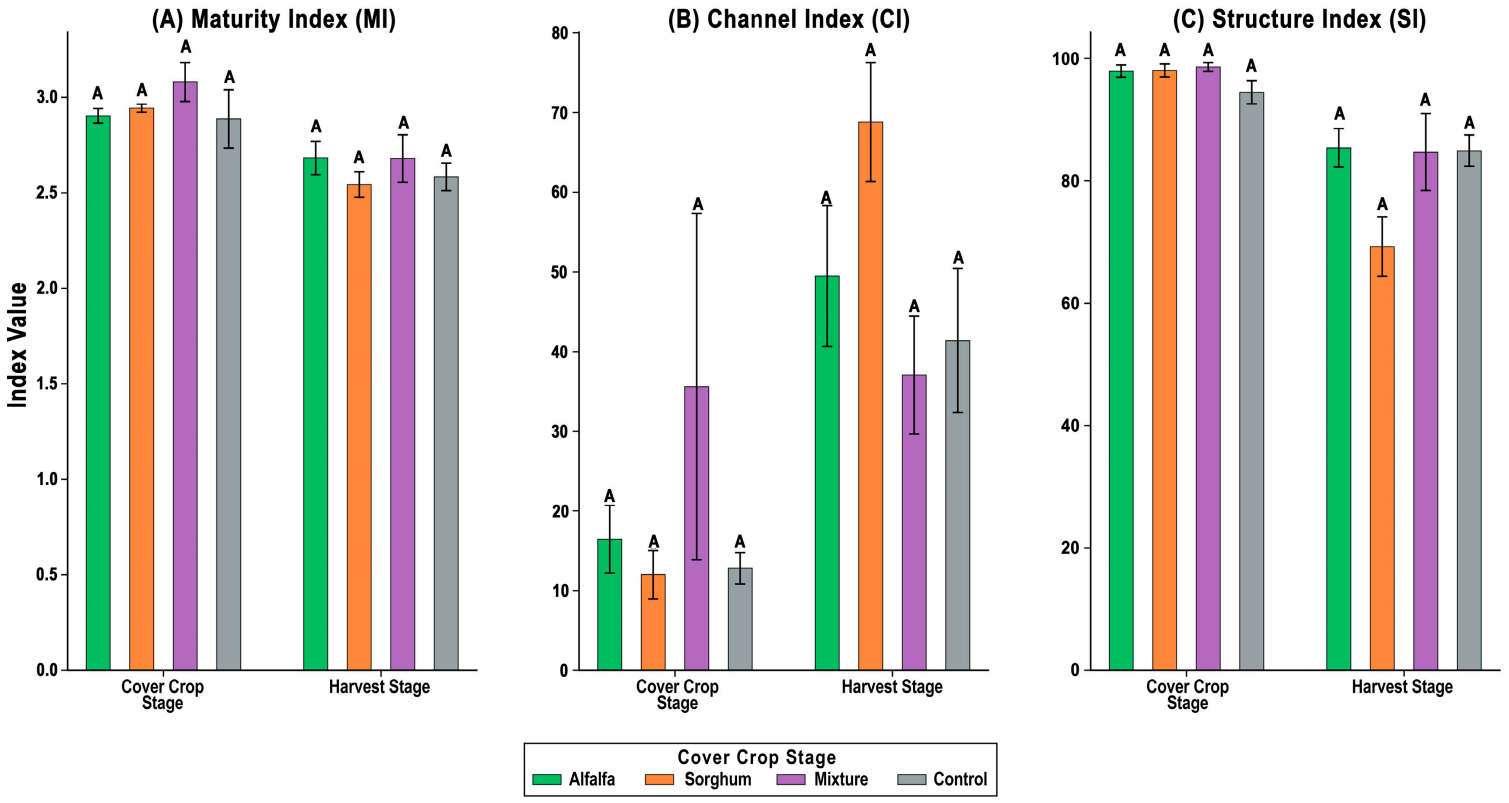
Nematode community ecological indices across cover crop treatments and sampling periods. (**A**) Maturity Index (MI); (**B**) Channel Index (CI); (**C**) Structure Index (SI). Bars represent mean ± SE for each cover crop treatment (alfalfa, sorghum, mixture, control) at the cover crop stage and harvest. MI reflects the weighted mean colonizer–persister (c-p) value of free-living nematodes [11]; CI indicates the relative contribution of fungal versus bacterial decomposition pathways [10]; SI reflects food web complexity and higher trophic connectivity. Letters denote statistical groupings among the cover crop treatments within each sampling time, based on one-way ANOVA followed by Tukey HSD post hoc test (α = 0.05); treatments sharing the same letter within a sampling time are not significantly different. The dominant effect was temporal where all three indices shifted significantly from cover crop stage to harvest (MI: F_1,44_ = 23.43, *p* < 0.001; CI: F_1,44_ = 17.07, *p* < 0.001; SI: F_1,44_ = 23.07, *p* < 0.001; Table S3), indicating seasonal food web restructuring across all treatments. Full ANOVA results including nitrogen and interaction effects are reported in Table S3.

Ecological indices revealed highly significant temporal shifts in food web condition from the cover crop stage to harvest (Figure 4). The Maturity Index (MI) declined significantly across all treatments (*p* < 0.001, partial η^2^ = 0.348), decreasing from 2.95 at the cover crop stage to 2.62 at harvest, indicating the increasing dominance of colonizer taxa during the growing season. The channel index (CI) increased significantly (*p* < 0.001, partial η^2^ = 0.279), rising from 14.3 at the cover crop stage to 49.1 at harvest, indicating a greater contribution of fungal decomposition pathways by harvest. Although communities remained predominantly bacterial-dominated at both timepoints (CI < 50), the pronounced rise in CI reflects seasonal restructuring of the soil food web as cover crop residues decomposed. The structure index (SI) also declined significantly (*p* < 0.001, partial η^2^ = 0.344), decreasing from 97.5 to 83.4, indicating reduced food web complexity and trophic connectivity by harvest. All individual ecological index values (MI, EI, SI, BI, CI) across treatments, sampling times, and nitrogen regimes are presented in Table S2. No significant cover crop, nitrogen, or cover crop × nitrogen interaction effects were detected for any community index (*p* > 0.05; Table S3), indicating that the seasonal food web restructuring occurred uniformly across all management treatments.

### 3.4. Plant-Parasitic Nematode Responses to Cover Crop Management

**Figure 5:**
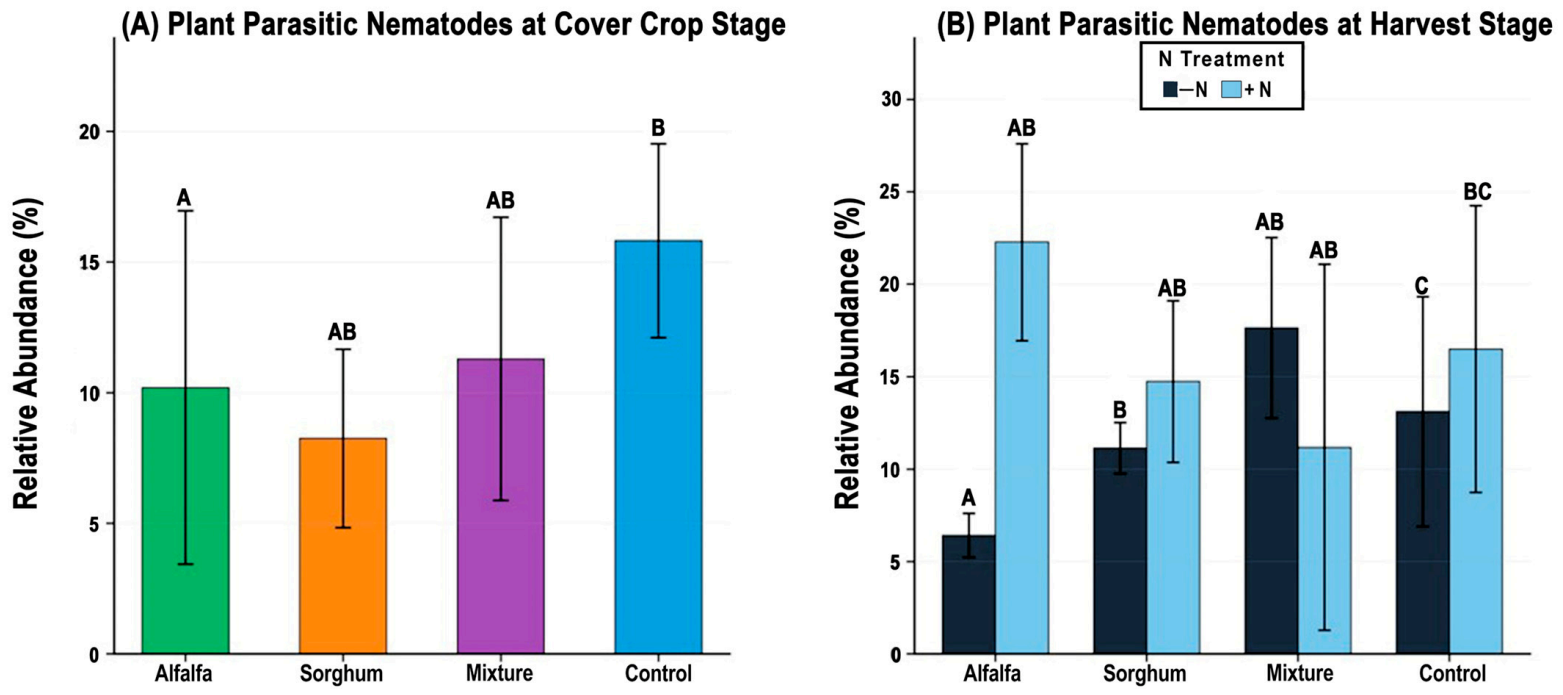
Relative abundance (±SE) of plant-parasitic nematodes across cover crop treatments. (**A**) Cover crop stage: letters denote statistical groupings among the cover crop treatments, based on one-way ANOVA followed by Tukey HSD post hoc test (α = 0.05); treatments sharing the same letter are not significantly different. Alfalfa plots showed the lowest plant-parasitic nematode abundance, significantly lower than control plots (*p* < 0.05). (**B**) Harvest: bars represent nitrogen treatment sub-groups (−N = no nitrogen; +N = with nitrogen). Letters denote statistical groupings among all cover crop × nitrogen treatment combinations, based on factorial ANOVA followed by Tukey HSD (α = 0.05); combinations sharing the same letter are not significantly different. Alfalfa without nitrogen maintained the lowest plant-parasitic nematode abundance, while control without nitrogen recorded the highest, highlighting a significant cover crop × nitrogen interaction (t = −2.91, *p* = 0.027; Table S1).

Cover crop treatments significantly influenced plant-parasitic nematode abundance across sampling periods (Figure 5). At the cover crop stage, alfalfa plots showed the lowest plant-parasitic nematode relative abundance (10.2 ± 3.5), while control plots had the highest (16.0%; *p* < 0.05). Sorghum (8.3%) and mixture (11.4%) plots occupied intermediate positions (Figure 5A). At harvest, a significant cover crop × nitrogen interaction was observed. Alfalfa without nitrogen maintained the lowest plant-parasitic nematode abundance across all treatment combinations (6.5%), while control without nitrogen recorded the highest (13.3%). However, the addition of nitrogen fertilizer records the most plant-parasitic nematode abundance, 22.0%, with alfalfa representing significant increase (*p* = 0.027). This interaction suggests that nitrogen fertilization counteracted the herbivore-suppressive effect of alfalfa residues, potentially by stimulating root growth, which in turn increased the availability of feeding sites for plant-parasitic nematodes (Figure 5B).

### 3.5. Beta-Diversity and Community Ordination

**Figure 6:**
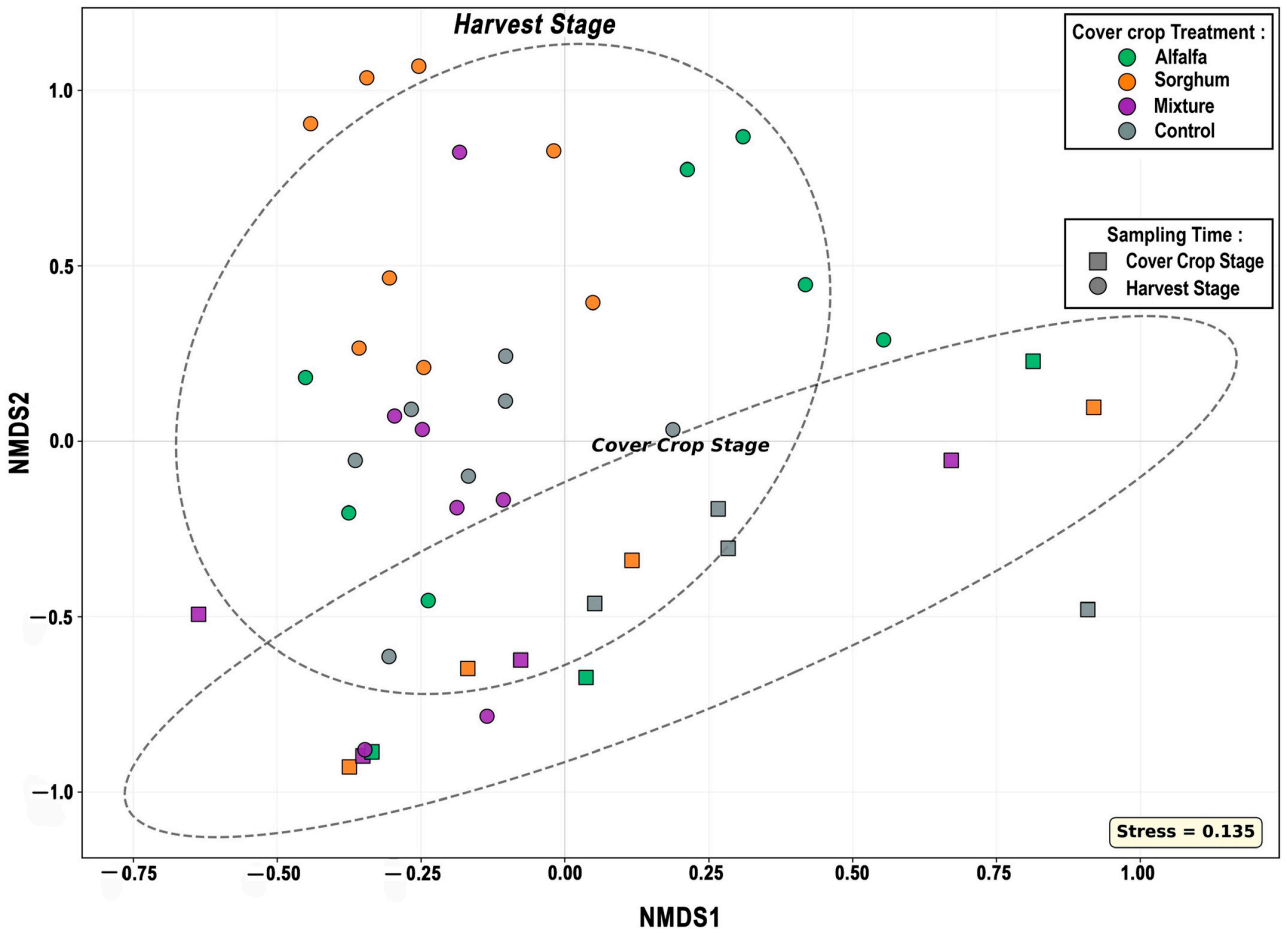
Non-metric multidimensional scaling (NMDS) ordination of soil nematode communities based on Bray–Curtis dissimilarity (stress = 0.135). Points represent individual soil samples color-coded by cover crop treatment (alfalfa, sorghum, mixture, control) and shaped by sampling time (squares = cover crop stage; circles = harvest). Dashed ellipses represent 95% confidence regions for each sampling period. Community composition was tested using PERMANOVA (9999 permutations) with sampling time, cover crop type, and nitrogen as factors. PERMANOVA confirmed significant effects of both sampling time (R^2^ = 0.167, *p* < 0.001) and cover crop type (R^2^ = 0.168, *p* = 0.004) on community composition; full PERMANOVA results including all factors and interactions are reported in Table S4.

Non-metric multidimensional scaling (NMDS) ordination based on Bray–Curtis dissimilarity (stress = 0.135) revealed distinct clustering patterns structured by both sampling time and cover crop treatment (Figure 6). Samples from the cover crop stage and harvest formed largely separate clusters along the ordination axes, with confidence ellipses showing minimal overlap between sampling periods. PERMANOVA confirmed statistically significant effects of both sampling time (R^2^ = 0.167, *p* < 0.001) and cover crop type (R^2^ = 0.168, *p* = 0.004) on nematode community composition, with the full model explaining 39.0% of total community variation (Table S4). Nitrogen fertilization did not significantly affect overall community composition (R^2^ = 0.011, *p* = 0.904), and no significant cover crop × sampling time interaction was detected (*p* = 0.430). PERMDISP confirmed homogeneous multivariate dispersions across all factor levels (*p* > 0.05), validating that the significant PERMANOVA results reflected genuine differences in community centroids rather than dispersion artifacts.

**Figure 7:**
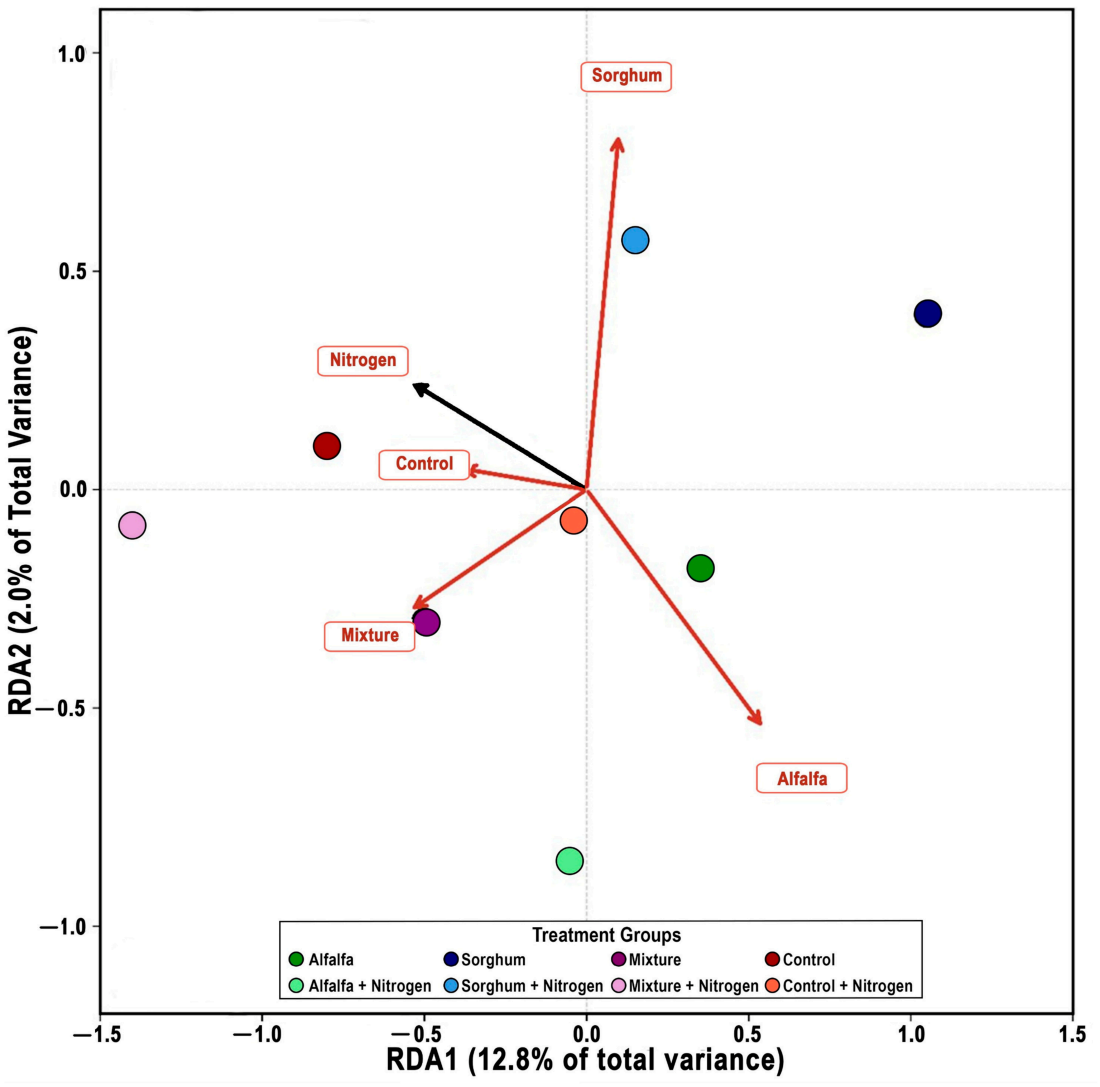
Redundancy analysis (RDA) biplot of soil nematode community composition at carrot harvest as a function of cover crop type and nitrogen fertilization. Red arrows represent cover crop type and black arrow represent nitrogen fertilizer; arrow length and direction indicate the strength and direction of association with the ordination axes. Points represent treatment group centroids for each cover crop × nitrogen combination. Cover crop type and nitrogen were fitted as constraining environmental variables, with significance assessed by permutation testing (9999 permutations). The overall RDA model explained 16.2% of total community variance (R^2^ = 0.162, *p* = 0.251); RDA1 captured 12.8% and RDA2 captured 2.0%. The overall model statistically showed that directional separation between alfalfa and sorghum along RDA1 was evident, while the mixture occupied an intermediate ordination position. It illustrates the directional relationships between environmental vectors and community centroids rather than pairwise treatment comparisons.

Redundancy analysis (RDA) at harvest revealed that cover crop and nitrogen treatments explained 16.2% of total community variance (R^2^ = 0.162, *p* = 0.251). Although the RDA model was not significant (*p* = 0.251), directional trends were evident: the sorghum vector showed the strongest positive projection along RDA1, consistent with its promotion of fungivore-dominated assemblages, while the alfalfa vector projected in the opposite direction along RDA2, reflecting its association with bacterivore-enriched communities. The mixture treatment occupied an intermediate ordination position, consistent with a blending of individual cover crop effects on the nematode community rather than a distinct community signature (Figure 7).

### 3.6. Feeding Group Inter-Correlations and Community Trade-Offs

**Figure 8:**
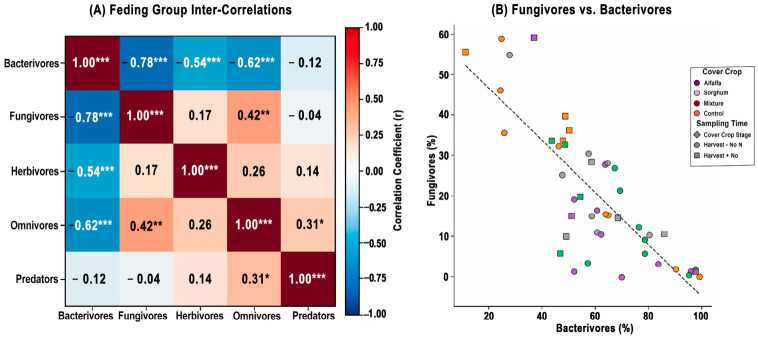
Nematode feeding group inter-correlations and community trade-offs. (**A**) Spearman rank correlation matrix among five nematode feeding groups. Samples were pooled across all cover crop treatments and sampling times; each cell represents the pairwise correlation between two feeding groups calculated across all samples. Color intensity and values indicate correlation strength; asterisks denote significance (* *p* < 0.05, ** *p* < 0.01, *** *p* < 0.001). Bacterivores showed strong negative correlations with fungivores (r = −0.78, *p* < 0.001), herbivores (r = −0.54, *p* < 0.001), and omnivores (r = −0.62, *p* < 0.001). (**B**) Scatter plot of fungivore versus bacterivore relative abundance, illustrating the dominant community trade-off (Spearman r = −0.777, *p* < 0.001). Points are color-coded by cover crop treatment and shaped by sampling time and nitrogen treatment. The dashed line represents the linear trend. No pairwise treatment comparisons are displayed; this figure illustrates ecological associations among feeding groups across the full dataset.

Spearman rank correlation analysis revealed strong and ecologically consistent associations among nematode-feeding groups (Figure 8A). Bacterivores showed significant negative correlations with fungivores (r = −0.78, *p* < 0.001), plant-parasitic nematodes (r = −0.54, *p* < 0.001), and omnivores (r = −0.62, *p* < 0.001), indicating that bacterivore dominance structurally excluded other feeding guilds. Fungivores and omnivores showed a significant positive association (r = 0.42, *p* < 0.01), while omnivores and predators were also positively correlated (r = 0.31, *p* < 0.05). The bacterivore–fungivore trade-off was the dominant community-structuring relationship, with a Spearman correlation of r = −0.777 (*p* < 0.001) across all samples (Figure 8B). This inverse relationship was expressed most strongly in sorghum harvest plots, where the shift from bacterivore-dominated to fungivore-dominated communities was most pronounced, while alfalfa plots consistently clustered in the high-bacterivore, low-fungivore region of the scatter plot. The mixture treatment exhibited an intermediate position on the bacterivore–fungivore gradient, positioning it as a balanced strategy that maintained moderate levels of both decomposition pathways without the extreme shifts observed in monoculture cover crop treatments.

### 3.7. Carrot Yield Responses

**Figure 9:**
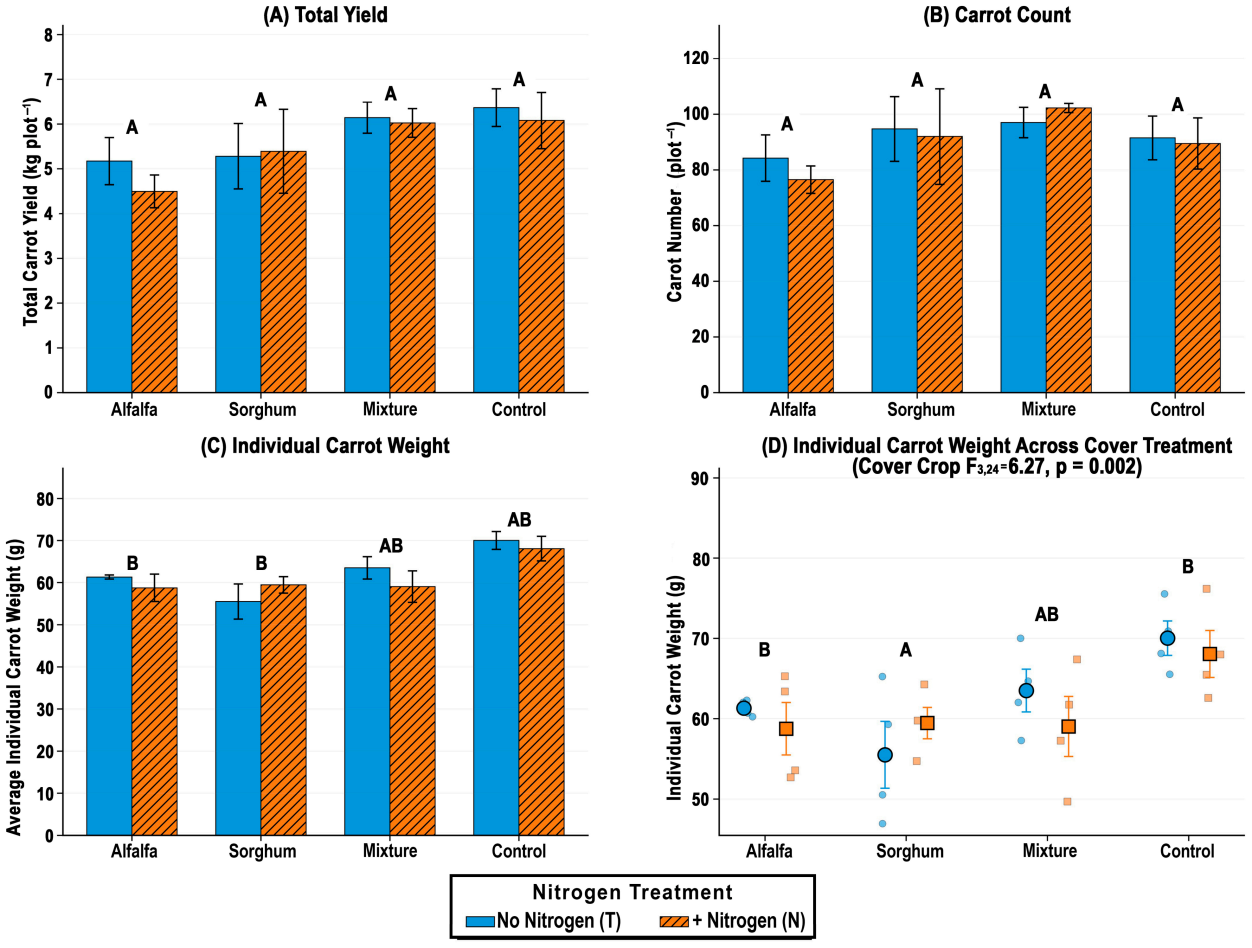
Carrot yield assessment across cover-crop treatments and nitrogen fertilization levels. (**A**) Total carrot yield (kg plot^−1^); (**B**) carrot count (number plot^−1^); (**C**) average individual carrot weight (g); (**D**) individual carrot weight distribution across cover crop treatments with jittered data points showing replicate-level variation. In (**A**–**C**), bars represent mean ± SE for no nitrogen (solid blue) and nitrogen fertilization (hatched orange). Large symbols in (**D**) represent treatment means ± SE; small symbols show individual replicates. Letters in all panels denote statistical groupings among the cover crop treatments based on two-way ANOVA (cover crop × nitrogen) followed by Tukey HSD (α = 0.05); treatments sharing the same letter are not significantly different. Cover crop type significantly affected individual carrot weight (panels C and D; F_3,24_ = 6.27, *p* = 0.002), with the mixture maintaining carrot weight comparable to the control while providing intermediate nematode management benefits. Total yield and carrot count remained consistent across all cover crop treatments (**A**,**B**). Full ANOVA partitioning for all yield parameters is reported in Table S1.

None of the treatments showed a significant difference between cover crop type and nitrogen fertilization with total carrot yield (*p* = 0.076) or carrot number per plot (*p* = 0.158) (Figure 9A,B). However, individual carrot weight was significantly influenced by cover crop type (*p* = 0.002). The highest individual carrot weight was observed in the cover crop mixture treatment (Figure 9C,D). These weight differences were consistent across nitrogen regimes, with no significant cover crop × nitrogen interaction within yield accumulation (*p* > 0.05). The inverse relationship between cover crop nematode-suppressive capacity (highest in alfalfa) and individual carrot weight (lowest in alfalfa) may suggest a potential trade-off between biological pest suppression and short-term yield performance that warrants further investigation across multiple growing seasons (Figure 9; Table S1B).

## 4. Discussion

This study demonstrates that cover crop functional type shapes soil nematode community composition and food web structure in carrot production systems. The observed treatment-specific responses likely reflect differences in residue quality, host suitability, and decomposition pathways associated with contrasting cover crop functional types. The dominance of bacterivores across all treatments (60.8% of total abundance) is consistent with patterns observed in annual cropping systems where bacterial decomposition pathways predominate [20]. These findings are consistent with the well-established predominance of bacterial decomposition pathways in intensively managed agricultural soils, where frequent disturbance and readily decomposable organic inputs favor bacterivore-dominated food webs [11]. Similar bacterivore dominance has been reported in spinach production systems under cover crop management [38] and in intensively managed vegetable cropping systems [39], confirming that this pattern extends beyond large-scale field crop systems to horticultural production contexts.

Alfalfa demonstrated the conditional suppression of plant-parasitic nematodes under nitrogen conditions, reducing *Pratylenchus* abundance to 6.5% at harvest compared to 13.3% in control plots; however, this benefit was entirely reversed when nitrogen was applied, with *Pratylenchus* abundance increasing to 22.0%. This nitrogen-dependent response suggests that alfalfa’s suppressive potential which may operate through non-host effects or legume-derived metabolites that favor bacterivore-dominated food webs is undermined by synthetic nitrogen inputs that stimulate carrot root growth and increase host availability. The substantial increase in fungivore abundance under sorghum treatment strongly supports the hypothesis that non-legume cover crops with high C:N ratio residues promote fungal decomposition pathways [19]. Sorghum produces abundant biomass with characteristically high C:N ratios [22] that favor fungal decomposer communities over bacteria [20]. This shift toward fungal-dominated decomposition is evidenced by the dramatic increase in fungivore populations, particularly *Aphelenchus*, which serve as reliable indicators of the fungal energy channel in soil food webs [39]. The pattern observed in our study is consistent with previous research demonstrating that organic amendments favor fungi and consequently fungivore nematodes [40,41,42]. The non-legume cover crops also promoted predator and omnivorous nematodes, which may subsequently enhance soil resilience and health in the long-term, in agreement with long-term cover crop experiments field crops such as corn and soybean production [43,44].

The mixture treatment, combining alfalfa and sorghum, exhibited intermediate effects on nematode community composition, reflecting the combined influence of legume and non-legume residue inputs. This intermediate positioning was further confirmed by redundancy analysis (RDA), where the mixture arrow occupied a position between the alfalfa and sorghum vectors, indicating that rather than creating a distinct community signature, the mixture treatment produced a blended community reflecting contributions from both individual cover crop components. Cover crop mixtures have been shown to optimize soil health benefits and pest suppression through diverse residue quality and functional traits [12,45,46], a pattern consistent with our observation that the mixture treatment supported more balanced fungivore-to-bacterivore ratios compared to single-species treatments, potentially indicating more diverse decomposition pathways operating simultaneously. This finding aligns with research demonstrating that higher cover crop diversity increases the abundance of bacterivores and fungivores relative to plant-parasitic nematodes [47,48] and is further supported by studies in orchard systems showing that diverse cover crop mixtures produce more balanced nematode community structures compared to single-species cover crops [49].

Among all three treatments that have been used in this study, alfalfa shows the highest suppression for plant-parasitic nematodes. This suppressive effect aligns with research demonstrating that alfalfa functions as a non-host or poor host for several economically important plant-parasitic nematodes [23,27]. Alfalfa has also been shown to promote microbiome-mediated suppression through the enrichment of beneficial soil bacteria enhancing plant immunity. Recent mechanistic studies have revealed that cover crop rotation primarily suppresses plant-parasitic nematodes by restructuring rhizosphere microbiota rather than through direct allelopathic effects or improved soil fertility alone [28]. This microbiome-mediated suppression may explain the sustained reduction in plant-parasitic nematode populations observed at both the cover crop stage and harvest. Comparable suppression of plant-parasitic nematodes by leguminous cover crops has been documented in carrot [27], tomato [50], and spinach [38] production systems, suggesting that this mechanism is broadly applicable across horticultural crops rather than being specific to the carrot system studied here.

However, nitrogen fertilization significantly reduced the suppressive effect of alfalfa on plant-parasitic nematodes at harvest (*p* = 0.027), with a 238% increase in their abundance when nitrogen was applied to alfalfa plots. The mechanism underlying this change may likely involve nitrogen-stimulated root growth and increased root exudate production, which would enhance host availability for obligate plant-parasitic genera, particularly *Pratylenchus*. Nitrogen addition has been shown to increase root biomass and alter soil faunal structure [51], thereby expanding the feeding substrate for root-lesion nematodes. Additionally, nitrogen fertilization may alter the composition of rhizosphere microbial communities that contribute to microbiome-mediated nematode suppression [28], effectively dismantling the biological suppression established by alfalfa residues. While this specific alfalfa × nitrogen interaction has not been previously documented in vegetable production systems, nitrogen-mediated shifts in nematode community composition have been reported across grassland and arable systems [52], with nitrogen addition consistently favoring colonizer-dominated, bacterivore-enriched communities through enhanced microbial activity and root biomass production. These findings collectively suggest that nitrogen management is a critical but underexplored variable in cover crop-based nematode suppression programs.

The decline in MI from 2.95 to 2.62 reflects a shift from persister-dominated toward colonizer-dominated communities over the cropping season, consistent with the progressive disturbance imposed by tillage, cover crop incorporation, and cash crop establishment. Similar temporal shifts in MI have been reported in cover-cropped tree systems [53] and across diverse agricultural management regimes [54], suggesting that this temporal pattern is a general feature of managed cropping systems rather than specific to carrot production.

The increase in channel index from 14.3 to 49.1 is particularly noteworthy, as it reflects a fundamental shift from bacterial-dominated (CI < 50) toward fungal-dominated (CI > 50, where 50 marks the bacterial–fungal balance point) decomposition pathways by harvest. This shift was most pronounced in sorghum plots, where CI reached 68.3 at harvest, consistent with sorghum residues favoring fungal decomposers [19,20]. The CI provides a mechanistic interpretation of the bacterivore–fungivore trade-off documented in Figure 8, confirming that this community-level trade-off reflects a genuine shift in the dominant decomposition channel rather than simply reflecting differential genus abundances. The CI has been identified as one of the most informative ecological indices for detecting cover crop effects on decomposition pathways in orchard systems [48] and in tree crop cover crop systems [53], supporting its utility in the vegetable production context examined here [38,55].

NMDS ordination and PERMANOVA confirmed that both sampling time and cover crop type play significant role in nematode community composition, together explaining 39.0% of total community variation. Sampling time accounted for a slightly greater proportion of explained variation (R^2^ = 0.167) than cover crop type (R^2^ = 0.168), indicating that temporal succession and cover crop identity contributed approximately equally to community differentiation [56]. The absence of a significant nitrogen effect on overall community composition (R^2^ = 0.011, *p* = 0.904) is consistent with the feeding group-level analyses, which showed that nitrogen primarily interacted with specific cover crop treatments (alfalfa) rather than exerting independent community-wide effects. This pattern differs from cereal crops ecosystem, where nitrogen addition typically produces stronger community-wide shifts [55,57], but is consistent with findings from annual vegetable production systems where cover crop identity rather than fertilization regime is the dominant structuring factor [58]. The RDA analysis at harvest, while not statistically significant overall (R^2^ = 0.162, *p* = 0.251), revealed informative directional trends among cover crop treatments. The separation of alfalfa and sorghum along RDA1 reflects their contrasting effects on decomposition pathways; bacterivore-promoting versus fungivore-promoting [44,59] while the intermediate positioning of the mixture treatment is consistent with a blending of these individual effects on the nematode community. The non-significance of the RDA model likely reflects the high within-treatment variability characteristic of metabarcoding data where spatial heterogeneity in soil nematode distributions can mask treatment effects at the community level [28,60].

The strong negative correlation between bacterivores and fungivores (r = −0.78, *p* < 0.001) represents the dominant community-structuring relationship in this system. This inverse relationship reflects the competitive exclusion between bacterial and fungal decomposition channels, a fundamental principle of soil food web ecology [61]. When bacterial decomposition predominates, fungal pathways are suppressed, and vice versa. The cover crop treatments effectively manipulated this balance: alfalfa promoted the bacterial channel (high bacterivores, low fungivores), while sorghum promoted the fungal channel. In contrast, the sorghum + alfalfa mixture maintained an intermediate position along the bacterivore–fungivore gradient, with bacterivore- and fungivore-relative abundances that fell between the extremes of the monoculture treatments. This intermediate positioning suggests that polyculture cover crop designs can moderate the decomposition pathway shifts induced by single-species cover crops, potentially providing greater stability in soil organic matter turnover and food web function across variable environmental conditions. This finding has practical implications for soil carbon management, as fungal-dominated food webs are associated with slower nutrient cycling but enhanced carbon stabilization, potentially contributing to soil carbon storage objectives [59]. The non-significant RDA result (*p* = 0.251) likely reflects the inherently high spatial heterogeneity of soil nematode distributions at the plot scale [62], a well-documented challenge in single-site field experiments with small plot sizes, which reduces statistical power and calls for the cautious interpretation of treatment effects, particularly those related to yield trade-offs that may not be reproducible across diverse agroecological contexts.

The absence of a statistically significant cover crop effect on total carrot yield (*p* = 0.076) suggests that, within a single growing season, the nematode community restructuring induced by cover crops may not translate into measurable yield benefits or penalties at the plot scale. This finding is consistent with reports that show nematode community shifts may require multiple growing seasons before the economically meaningful suppression of plant-parasitic species reduces crop damage sufficiently to influence yield [63], a pattern similarly observed in cover crop studies in carrot [64] and corn–soybean production systems [47], where single-season cover cropping produced measurable community-level changes without corresponding yield responses. However, the significant reduction in individual carrot weight under alfalfa and sorghum treatments compared to the mixture (69.15 g) and control warrants attention, as individual carrot size is an important commercial quality parameter [64]. Notably, the mixture maintained individual carrot weight higher than single-cover use while preserving intermediate nematode suppression benefits, positioning it as a pragmatic choice for growers seeking both soil health and yield objectives [64]. This finding reinforces the recommendation that functionally diverse cover crop mixtures, particularly legume–non-legume combinations [58], hold the greatest potential for Ontario carrot producers seeking to simultaneously optimize biological pest suppression, soil food web health, and agronomic performance, rather than relying on single-species cover crops which offer only partial benefits [64]. Furthermore, longer-term studies tracking nematode community changes over multiple cover crop cycles would clarify the cumulative effects of repeated cover cropping on soil food web development [63].

Our findings demonstrate the importance of considering cover crop functional diversity rather than individual species when designing biologically based soil health strategies. Although monoculture cover crops provided specific ecological benefits, combining functionally contrasting species produced a more balanced response across nematode community structure, soil food web condition, and carrot performance [65]. These results support the increasing interest in multifunctional cover crop systems for sustainable horticultural production [66]. This study was conducted under a single set of environmental and management conditions. Although this experimental design enabled the detailed characterization of treatment responses, future multi-site and multi-year studies would help determine the consistency of these patterns under a broader range of production environments.

## 5. Conclusions

This study demonstrates that cover crop functional type is a key driver of soil nematode community composition and soil food web dynamics in carrot production systems. Individual cover crops showed specific benefits: alfalfa suppressed plant-parasitic nematodes, but this effect was reduced by nitrogen fertilization, while sorghum promoted fungal decomposition pathways offering limited pest suppression. Nematode community ecological indices (MI, CI, SI) revealed highly significant seasonal food web restructuring after a single-cover crop cycle. The legume–non-legume mixture emerged as the most agronomically balanced treatment, uniquely combining moderate nematode suppression with the highest individual carrot, and the highest Structure Index trending towards better soil health. These findings position functionally diverse cover crop mixtures as having the greatest potential for carrot growers seeking to simultaneously optimize soil biological health, pest suppression, and crop quality. Beyond demonstrating treatment effects on nematode communities, this study showed the value of considering cover crop functional diversity as a strategy for improving multiple components of agroecosystem performance simultaneously. Rather than maximizing a single outcome, functionally diverse cover crop mixtures may help balance biological pest suppression, soil food web health, and crop productivity. Future multi-year studies should examine whether repeated cover cropping with diverse mixtures could better transition food web condition and elucidate the microbial mechanisms underlying the soil health improvement.

## Data Availability

The raw sequencing datasets generated and analyzed during the current study are available in the NCBI Sequence Read Archive (SRA) repository under BioProject accession number PRJNA1492785 (https://www.ncbi.nlm.nih.gov/bioproject/PRJNA1492785).

## Supplementary Materials

The following supporting information can be downloaded at: https://www.mdpi.com/article/10.3390/biology15151226/s1, Figure S1. Schematic diagram of the split-plot experimental design established at Jordan Station, Ontario, Canada (43.16° N, 79.36° W). The experiment comprised four complete replications (Rep 1–Rep 4) arranged within a 25 m × 20 m field area. Cover crop treatments were assigned to main plots: Sorghum (Red border); Alfalfa (Yellow border), Sorghum+Alfalfa mixture (Blue border), Nitrogen were assigned to sub-plots (black), and no cover crop control (Light green border); Table S1. Statistical outputs for nematode feeding group relative abundances and carrot yield parameters across cover crop type, nitrogen fertilization, and sampling time, (A) Nematode Feeding Group Relative Abundances, (B) Carrot Yield Parameters; Table S2. Nematode community ecological indices Mean (±SE) across cover crop treatments and nitrogen regimes at the cover crop stage and at harvest; Table S3. Nematode community ecological indices across cover crop type, nitrogen fertilization, and sampling time in a carrot production system; Table S4. Permutational multivariate analysis of variance (PERMANOVA) of Bray-Curtis dissimilarity matrices testing the effects of sampling time, cover crop type, and nitrogen fertilization on soil nematode community composition in a carrot pro-duction system.
