# Supplementary material for: Metabarcoding Reveals Cover Crop Functional Type as Primary Driver of Soil Nematode Community Composition in a Carrot Production System
Source: Biology (Basel). 2026 Jul 23;15(15):1226. doi: 10.3390/biology15151226 (PMC13464887; doi:10.3390/biology15151226)
Supplement: Supplementary file 1 [file biology-15-01226-s001.zip › Supplementary figure__Manuscript ID biology-4219415.pdf]

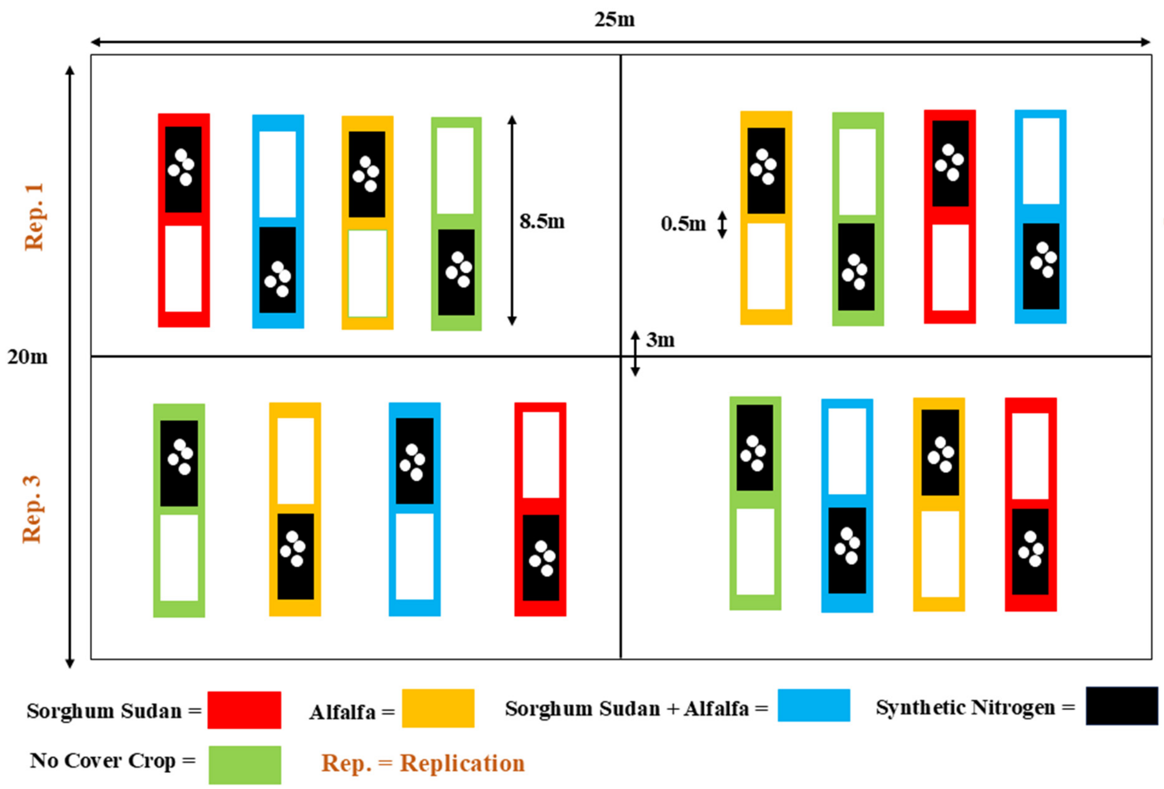

Supplementary Figure S1. Schematic diagram of the split-plot experimental design established at Jordan Station, Ontario, Canada (43.16° N, 79.36° W). The experiment comprised four complete replications (Rep 1–Rep 4) arranged within a 25 m × 20 m field area. Cover crop treatments were assigned to main plots: Sorghum (Red border); Alfalfa (Yellow border), Sorghum+Alfalfa mixture (Blue border), Nitrogen were assigned to sub-plots (black), and no cover crop control (Light green border).
