# Supplementary material for: Metabarcoding Reveals Cover Crop Functional Type as Primary Driver of Soil Nematode Community Composition in a Carrot Production System
Source: Biology (Basel). 2026 Jul 23;15(15):1226. doi: 10.3390/biology15151226 (PMC13464887; doi:10.3390/biology15151226)
Supplement: Supplementary file 1 [file biology-15-01226-s001.zip › Supplementary Tables_Manuscript ID biology-4219415.pdf]

Table S1. Statistical outputs for nematode feeding group relative abundances and carrot yield parameters across cover crop type, nitrogen fertilization, and sampling time

(A) Nematode Feeding Group Relative Abundances

Feeding Group	Fixed Effect	df (num, den)	F-value	p-value	Partial η^2	Sig.
**Bacterivores**	Cover Crop	3, 42	1.86	0.152	0.117	ns
	Sampling Time	1, 44	9.84	0.003	0.183	**
	Nitrogen	1, 28	0.08	0.781	0.003	ns
	CC × N (Harvest)	3, 22	2.34	0.101	0.242	ns
**Fungivores**	Cover Crop	3, 42	2.92	0.045	0.172	*
	Sampling Time	1, 44	12.39	0.001	0.220	***
	Nitrogen	1, 28	0.01	0.911	0.000	ns
	CC × N (Harvest)	3, 22	0.89	0.460	0.109	ns
**Plant Parasitic Nematodes**	Cover Crop	3, 42	0.29	0.832	0.020	ns
	Sampling Time	1, 44	0.86	0.360	0.019	ns
	Nitrogen	1, 28	1.45	0.239	0.049	ns
	CC × N (Harvest)	3, 22	1.41	0.266	0.161	ns
	Alfalfa × N	1, 6	t = −2.91	0.027	—	*
**Omnivores**	Cover Crop	3, 42	0.96	0.419	0.064	ns
	Sampling Time	1, 44	0.00	0.972	0.000	ns
	Nitrogen	1, 28	2.77	0.107	0.090	ns
	CC × N (Harvest)	3, 22	2.43	0.092	0.249	ns

(B) Carrot Yield Parameters

Yield Parameter	Source	df	SS	MS	F-value	p-value	Partial η^2
**Total Yield (kg plot^{-1})**	Cover Crop	3	3.84	1.280	2.60	0.076	0.245
	Nitrogen	1	0.18	0.180	0.36	0.553	0.015
	CC × N	3	0.25	0.083	0.17	0.917	0.021
	Residual	21	11.82	0.563	—	—	—
**Individual Weight (g)**	Cover Crop	3	1,240	413.3	6.00	0.003	0.429
	Nitrogen	1	26.9	26.9	0.39	0.539	0.016
	CC × N	3	148.2	49.4	0.72	0.552	0.082
	Residual	21	1,653	68.9	—	—	—
**Carrot Number (plot^{-1})**	Cover Crop	3	842	280.7	1.89	0.158	0.213
	Nitrogen	1	35	35.0	0.24	0.627	0.011
	CC × N	3	183	61.0	0.41	0.748	0.055
	Residual	21	3,121	148.6	—	—	—

(A): Cover crop type, sampling time, nitrogen fertilization, and their interactions were tested using one-way ANOVA or factorial ANOVA as appropriate. Sampling Time compares Cover crop stage vs. Harvest (df = 1). Nitrogen effect and CC × N interaction tested at Harvest only (n = 30). Alfalfa × N tested by independent t-test. Partial η^2 = SS_effect / (SS_effect + SS_residual). (B): Cover crop type and nitrogen were fixed factors; Tukey HSD for individual carrot weight: Control (a) > Mixture (ab) > Alfalfa (b) = Sorghum (b). Significance: $p < 0.001$; $p < 0.01$; $p < 0.05$; ns = not significant.

Table S2. Nematode community ecological indices Mean (± SE) across cover crop treatments and nitrogen regimes at the cover crop stage and at harvest

Stage	Treatment	Nitrogen	MI	EI	SI	BI	CI
Cover Crop Stage	Alfalfa	—	2.90 ± 0.04	84.4 ± 2.8	98.0 ± 1.0	1.7 ± 0.8	15.9 ± 4.4
	Sorghum	—	2.94 ± 0.03	90.0 ± 2.4	98.7 ± 0.7	1.2 ± 0.6	9.5 ± 1.9
	Mixture	—	3.07 ± 0.10	79.4 ± 10.1	98.9 ± 0.6	1.1 ± 0.5	33.4 ± 22.3
	Control	—	2.88 ± 0.16	87.5 ± 4.2	95.8 ± 2.0	3.3 ± 1.5	9.9 ± 1.9
Harvest	Alfalfa	No Nitrogen	2.85 ± 0.07	60.2 ± 4.0	91.0 ± 2.9	7.8 ± 2.4	63.0 ± 11.4
	Alfalfa	With Nitrogen	2.55 ± 0.13	71.6 ± 4.7	81.1 ± 4.3	12.6 ± 2.9	34.9 ± 9.3
	Sorghum	No Nitrogen	2.65 ± 0.09	56.2 ± 2.6	72.3 ± 5.5	20.2 ± 3.2	72.9 ± 9.0
	Sorghum	With Nitrogen	2.44 ± 0.07	60.1 ± 4.9	66.4 ± 8.6	22.5 ± 5.4	64.5 ± 12.9
	Mixture	No Nitrogen	2.71 ± 0.16	71.7 ± 3.9	88.9 ± 4.5	7.6 ± 2.6	35.6 ± 4.9
	Mixture	With Nitrogen	2.64 ± 0.24	64.4 ± 3.2	79.3 ± 14.4	12.6 ± 7.5	50.5 ± 6.1
	Control	No Nitrogen	2.47 ± 0.00	73.0 ± 2.3	80.6 ± 1.2	12.7 ± 1.1	34.6 ± 4.3
	Control	With Nitrogen	2.67 ± 0.11	69.2 ± 7.7	88.8 ± 3.3	8.3 ± 2.4	45.6 ± 16.2

Values are mean ± standard error. MI = Maturity Index; EI = Enrichment Index; SI = Structure Index; BI = Basal Index; CI = Channel Index. Cover Crop Stage = post-incorporation sampling; Harvest = end-of-season sampling. — = nitrogen sub-treatment not applied at cover crop stage. n = 4 plots per Treatment × Nitrogen combination. Indices calculated following Bongers (1990) and Ferris et al. (2001). All indices showed highly significant temporal (Stage) effects ($p < 0.001$)

Table S3. Nematode community ecological indices across cover crop type, nitrogen fertilization, and sampling time in a carrot production system

Community Index	Fixed Effect	df (num, den)	F-value	p-value	Partial η^2
**Maturity Index (MI)**	Cover Crop	3, 42	0.68	0.569	0.046
	Sampling Time	1, 44	23.43	<0.001	0.348
	Nitrogen	1, 28	1.77	0.195	0.059
	CC × N (Harvest)	3, 22	1.63	0.211	0.182
**Channel Index (CI)**	Cover Crop	3, 42	0.94	0.432	0.063
	Sampling Time	1, 44	17.07	<0.001	0.279
	Nitrogen	1, 28	0.05	0.818	0.002
	CC × N (Harvest)	3, 22	2.10	0.130	0.222
**Structure Index (SI)**	Cover Crop	3, 42	1.89	0.147	0.119
	Sampling Time	1, 44	23.07	<0.001	0.344
	Nitrogen	1, 28	0.84	0.368	0.029
	CC × N (Harvest)	3, 22	0.97	0.427	0.116
**Basal Index (BI)**	Cover Crop	3, 42	2.38	0.083	0.145
	Sampling Time	1, 44	27.07	<0.001	0.381
	Nitrogen	1, 28	0.39	0.540	0.014
	CC × N (Harvest)	3, 22	0.73	0.544	0.091
**Enrichment Index (EI)**	Cover Crop	3, 42	0.88	0.459	0.059
	Sampling Time	1, 44	17.99	<0.001	0.290
	Nitrogen	1, 28	1.53	0.226	0.052
	CC × N (Harvest)	3, 22	0.23	0.877	0.030

Nematode community indices were calculated following Bongers (1990) and Ferris et al. (2001). MI = Maturity Index, reflecting the weighted mean colonizer–persister (c-p) value of free-living nematodes (higher values indicate more mature, stable food webs). CI = Channel Index, indicating the relative contribution of fungal vs. bacterial decomposition pathways (CI > 50 = fungal-dominated; CI < 50 = bacterial-dominated). SI = Structure Index, reflecting food web complexity and trophic chain length. BI = Basal Index, representing the proportion of basal (opportunistic) taxa in the community. EI = Enrichment Index, indicating resource availability and nutrient enrichment status. Sampling Time compares Cover Crop Stage vs. Harvest (df = 1). Nitrogen effect and CC × N interaction tested at Harvest only (n = 30). Partial η^2 = SS_effect / (SS_effect + SS_residual). All indices showed highly significant temporal effects ($p < 0.001$), indicating strong seasonal shifts in food web condition from cover crop incorporation to carrot harvest. No significant cover crop, nitrogen, or interaction effects were detected for any index. Significance: $p < 0.001$; $p < 0.01$; $p < 0.05$; ns = not significant.

Table S4. Permutational multivariate analysis of variance (PERMANOVA) of Bray-Curtis dissimilarity matrices testing the effects of sampling time, cover crop type, and nitrogen fertilization on soil nematode community composition in a carrot pro-duction system

Factor	df	SS	R^2	F-value	p-value
Sampling Time	1	1.258	0.167	8.851	0.0002
Cover Crop Type	3	1.261	0.168	2.823	0.004
Nitrogen Fertilization	1	0.042	0.011	0.303	0.904
CC × Sampling Time	3	0.372	0.050	1.019	0.430
Residuals	37	4.580	0.610	—	—
Total	45	7.513	1.000	—	—

R^2 represents the proportion of total Bray-Curtis dissimilarity variance attributable to each factor. The full model explains 39.0% of total community variation, with 61.0% attributable to within-treatment heterogeneity. PERMDISP tests confirmed homogeneous multivariate dispersions across all factor levels ($p > 0.05$ for all factors), validating that significant PERMANOVA results reflect genuine differences in community composition centroids. Significance: *** $p < 0.001$; ** $p < 0.01$; * $p < 0.05$; ns = not significant.
